# Phylogeography and Genetic Diversity in a Southern North American Desert: *Agave kerchovei* From the Tehuacán-Cuicatlán Valley, Mexico

**DOI:** 10.3389/fpls.2020.00863

**Published:** 2020-06-23

**Authors:** Erika Aguirre-Planter, J. Gilberto Parra-Leyva, Santiago Ramírez-Barahona, Enrique Scheinvar, Rafael Lira-Saade, Luis E. Eguiarte

**Affiliations:** ^1^Laboratorio de Evolución Molecular y Experimental, Departamento de Ecología Evolutiva, Instituto de Ecología, Universidad Nacional Autónoma de México, Mexico City, Mexico; ^2^Departamento de Botánica, Instituto de Biología, Universidad Nacional Autónoma de México, Mexico City, Mexico; ^3^Unidad de Biotecnología y Prototipos, Facultad de Estudios Superiores Iztacala, Universidad Nacional Autónoma de México, Mexico City, Mexico

**Keywords:** Tehuacán-Cuicatlán, *Agave*, species distribution models, incipient speciation, population genetics, Pleistocene, endemic

## Abstract

The Tehuacán-Cuicatlán Valley, located at the southeast of the state of Puebla and the northeast of the state of Oaxaca in Central Mexico, south of the Trans-Mexican Volcanic Belt (TMVB), is of particular interest for understanding the evolutionary dynamics of arid and semi-arid environments, being one of the main reservoirs of biological diversity for the arid zones of North America, including the highest diversity of Agavaceae worldwide and high levels of endemism. Studying in detail the phylogeography, environmental history and population genetics of representative species will hopefully shed light on the evolutionary and ecological dynamics that generated the tremendous biodiversity and endemism of this important region in Mexico. We sequenced three non-coding regions of chloroplast genome of *Agave kerchovei*, a representative species of the Tehuacán Valley, generating 2,188 bp from 128 individuals sampled from eight populations throughout the species range. We used this data set to (i) characterize the levels of genetic diversity and genetic structure in *A. kerchovei*; (ii) predict the distribution of *A. kerchovei* for the present day, and to reconstruct the past geographical history of the species by constructing ecological niche models (ENM); and (iii) compare the levels of diversity in this species with those estimated for the widely distributed *Agave lechuguilla*. *Agave kerchovei* has high levels of total chloroplast genetic variation (*Hd* = 0.718), especially considering that it is a species with a very restricted distribution. However, intrapopulation diversity is low (zero in some populations), and genetic structure is high (*F*_ST_ = 0.928, *G*_ST_ = 0.824), which can be expected for endemic species with isolated populations. Our data suggest that Pleistocene glacial cycles have played an important role in the distribution of *A. kerchovei*, where the climatic variability of the region – likely associated with its topographic complexity – had a significant effect on the levels of genetic diversity and population dynamics, while the potential distribution of the species seems to be stable since the middle Holocene (6 kya). We conclude that in *A. kerchovei* there is a core group of populations in the Tehuacán Valley, and peripheric populations that appear to be evolving independently and thus the species is fundamentally an endemic species from the Tehuacán Valley while the populations outside the Valley appear to be in the process of incipient speciation.

## Introduction

The Pleistocene glacial periods have been major factors influencing the geographical distribution, demographic dynamics and patterns of genetic diversity of many species (Haffer and Prance, 2001; Hewitt, 2004; Stewart et al., 2010; Ramírez-Barahona and Eguiarte, 2013; Castellanos-Morales et al., 2016). During the Pleistocene pronounced glacial cycles, temperature fluctuations have been very marked, alternating between a cooler climate than the present (6 to 8°C lower) in the glacial periods and a warmer climate than the present (2 to 3°C higher) in the interglacials (Caballero et al., 2010; Ramírez-Barahona and Eguiarte, 2013). Throughout the repeated Pleistocene cycles, populations of warm desert biota retreated from northern to southern latitudes during adverse environmental conditions, and then recolonized or expanded towards the north when conditions improved (Hewitt, 2004; Ramírez-Barahona and Eguiarte, 2013; Loera et al., 2017; Scheinvar et al., 2017). This occurred several times, causing cycles of range contraction and expansion. After the Last Glacial Maximum (LGM ∼ 23–18 kya), different areas of Central and North America became more arid (Ramírez-Barahona and Eguiarte, 2013) and this aridification probably led to important changes in the distribution and composition of species in arid zones.

**FIGURE 1:**
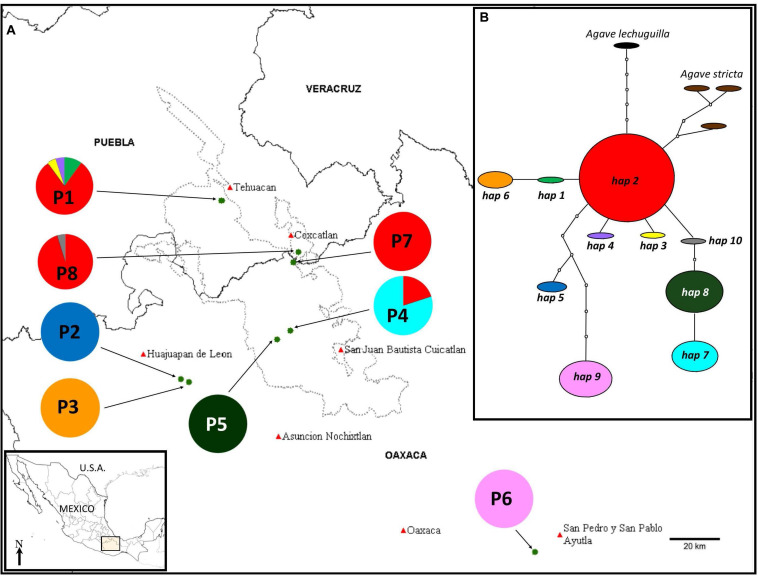
**(A)** Population locations (names as in Table 1) and distribution of haplotypes in the different populations. **(B)** Minimum-spanning network of the chloroplast haplotypes found in the eight populations of *A. kerchovei* studied. The network represents the most parsimonious connections. Each haplotype is represented by a circle whose surface is proportional to the number of individuals bearing it. Lines between haplotypes indicate one mutational change. Missing haplotypes in the sample are represented by dotted circles. Populations 1, 7, and 8 correspond to one SAMOVA group, while each of the rest of the populations represent one different group, with a total of six groups, *F_CT_* = 0.915.

Of particular interest for understanding the evolutionary dynamics of arid and semiarid environments in North America is the Valley of Tehuacán-Cuicatlán Biosphere Reserve (called Tehuacán Valley hereafter) (Dávila et al., 2002; Valiente-Banuet et al., 2009). The Tehuacán Valley is located at the southeast of the state of Puebla and the northeast of the state of Oaxaca in Central Mexico, south of the Trans-Mexican Volcanic Belt (TMVB), representing the southernmost semiarid area in Mexico (Rzedowski and Huerta, 1978; Hafner and Riddle, 2011; El-Ghani et al., 2017) (Figure 1A). It has a distinctive biotic megadiversity and it is one of the main reservoirs of biological diversity for the arid zones of North America. Despite being geographically isolated from the rest of the arid and semiarid regions of North America (e.g., Chihuahuan and Sonoran deserts), the Tehuacán Valley has been characterized as a hot-spot of plant diversity and endemism (UNESCO, 2012), with more than 3,000 species of seed plants reported (Dávila et al., 2002). According to Valiente-Banuet et al. (2009) the late Pleistocene climate changes (10,000 to 1,000 kya) were very important for the current geomorphic and biotic composition of the Tehuacán Valley, suggesting that local plant communities were recently assembled.

Considering that the total area of the Tehuacán Valley is relatively small (10,000 km^2^) and that the current vegetation of the area is of recent origin (Valiente-Banuet et al., 2009), it is interesting that the Valley harbors the highest diversity of succulent plant families such as Cactaceae and Agavaceae (Valiente-Banuet et al., 2000; Tambutti, 2002; García-Mendoza, 2011; Delgado-Lemus et al., 2014). In addition, this region comprises an heterogeneous mosaic of environments determined by climates, soils, geomorphology and elevation, which is reflected in nearly 36 types of plant associations (i.e., groups of plant species that occur across the landscape; Valiente-Banuet et al., 2000, 2009), and in a high degree of endemism for several different groups (Dávila et al., 2002). In particular, the Tehuacán Valley contains the highest richness of *Agave* species in the world (Tambutti, 2002; García-Mendoza, 2011). A total of 34 *Agave* species have been recorded, 25 of them native to the region, while seven of them are endemic to this area (García-Mendoza, 2011; Delgado-Lemus et al., 2014). In comparison, in the Chihuahuan Desert, the largest and warmest desert in North America (Hernández et al., 2001; Olson and Dinerstein, 2002; Scheinvar et al., 2017), with an area of approximately 507,000 km^2^ (Granados-Sánchez et al., 2011) only 19 *Agave* species have been reported (Chihuahuan Desert Homepage, Centennial Museum, The University of Texas^1^).

We propose that by studying in detail the phylogeography of representative species in the Tehuacán Valley we will be able to better understand the evolutionary dynamics that generate the high biodiversity and endemism found in this semiarid regions. Accordingly, we investigated the population structure and phylogeography of *Agave kerchovei* Lem., examining polymorphisms observed in cpDNA sequences. This species is representative of the Tehuacán Valley, given that it is mostly restricted to this region (García-Mendoza, 2011; Brena-Bustamante, 2012; Brena-Bustamante et al., 2013) (Figure 1A). Our study aimed to (i) characterize the levels of genetic diversity and genetic structure in *A. kerchovei*; (ii) predict the distribution of *A. kerchovei* for the present day and reconstruct the past geographical history of the species, by constructing ecological niche models (ENM); and (iii) compare the levels of diversity in this species with those estimated for the widely distributed *A. lechuguilla*, a related species from the Chihuahuan desert. We expected to detect low levels of genetic diversity given the small size and restricted distribution of *A. kerchovei* populations, as well as a high genetic structure, given population isolation. Due to the high topographic complexity of the Tehuacán Valley (Valiente-Banuet et al., 2000, 2009), which translates into high climatic variability, we also predicted that the high environmental variance among populations will translate into high levels of genetic differentiation in *A. kerchovei* compared to the populations of the closely related but widespread *A. lechuguilla*, endemic to the Chihuahuan Desert (Scheinvar et al., 2017).

## Materials and Methods

### Study Species

In this study, we will follow Gentry’s classification (1982), who does not recognize subspecies or varieties of *A. kerchovei* [although The Standard Cyclopedia of Horticulture (1914) mentions varieties of *A. kerchovei*, including *A. kerchovei* var. *beaucarnei*, also included in the Tropicos Database^2^, these varieties are now regarded as synonyms of *A. kerchovei* (The Plant List^3^; and Global Biodiversity Information Facility, GBIF^4^)].

**FIGURE 2:**
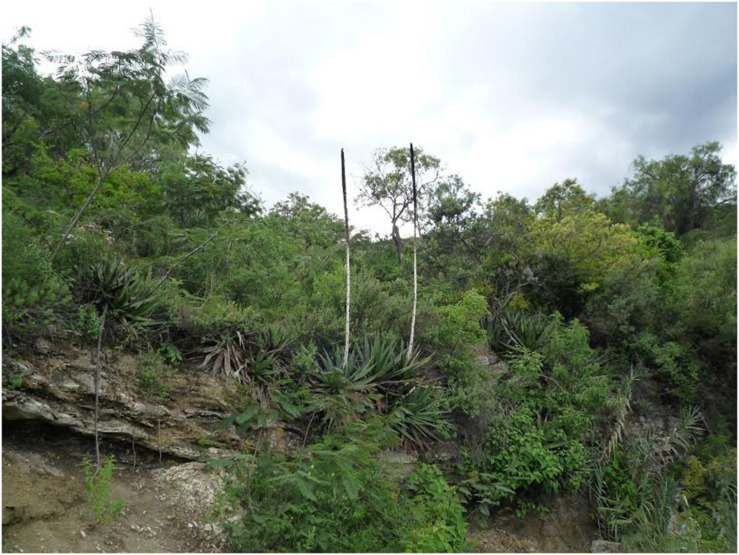
*Agave kerchovei* from Villa Tamazulapám del Progreso, Oaxaca (P2 in Figure 1).

*Agave kerchovei* is a species with a narrow distribution. Little is known about its natural history or ecology (Brena-Bustamante, 2012; Brena-Bustamante et al., 2013). Gentry(1982, p. 153; 190), reports the species distribution in the states of Puebla and Oaxaca and in one locality in the state of Hidalgo (Barranca de Metztitlán). More recently, García-Mendoza (2011) reported populations only in the states of Oaxaca and Puebla and extensive field work with *Agave* in the Barranca de Metztitlán (Hidalgo) did not detect a single plant of the species (Rocha et al., 2005; Eguiarte and Scheinvar, 2008). *Agave kerchovei’s* present distribution is mostly restricted to the Tehuacán Valley (García-Mendoza, 2011; Brena-Bustamante, 2012) (Figure 1A), although more populations have been reported in herbarium records (see below). It is currently considered as a vulnerable species in the IUCN Red List of Threatened Species 2019 (García-Mendoza et al., 2019). Furthermore, the known populations of *A. kerchovei* are small and isolated, sometimes consisting of fewer than 10 individuals (Brena-Bustamante, 2012), inhabiting rough terrain within the mountainous regions of the Valley. *Agave kerchovei* is a medium-sized *Agave* with a brilliant green short stem and wide rosettes (Gentry, 1982) (Figure 2), and evidence of both sexual and asexual reproduction has been observed in some of its populations (Brena-Bustamante, 2012). Local people sometimes promote *in situ* local propagation of propagules of the species (Delgado-Lemus et al., 2014), collecting the flowers for human consumption and their leaves for making fences (Brena-Bustamante, 2012; Brena-Bustamante et al., 2013).

### Population Sampling

Individuals from eight wild populations were sampled across the species’ geographical range throughout the Valley and nearby areas (Figure 1 and Table 1). For each population, leaf tissue from 13–23 adult individuals, separated by at least 5 m to avoid sampling the same genetic clone, were collected, with the exception of populations 2 and 3, where only four and eight adult individuals were found and collected, respectively (Table 1). Leaf tissue of each collected individual was stored at −80°C in the tissue collection of the Instituto de Ecología, UNAM, and is available upon request. A voucher specimen of population 8 (San Rafael Coxcatlán) (collection number, 117) is deposited at the Facultad de Estudios Superiores, Iztacala, UNAM herbarium, IZTA (acronym fide Thiers, 2016). Additionally, herbarium samples for different localities are deposited at the MEXU, UNAM herbarium.

**TABLE 1:** Measures of genetic variation for the analyzed populations of *Agave kerchovei*.

Population	Locality	Latitude (N)	Longitude (W)	Altitude (m)	*N*	*S*	*h*	*Hd*	π	*D’* Tajima
1	SW from Santa María Coapan, Puebla	18° 24′	97° 26′	1817	20	3	4	0.36316	0.00018	−1.4407
2	NW from Villa de Tamazulapam del Progreso, Oaxaca	17° 42′	97° 37′	1927	4	0	1	0	0	0
3	NW from Villa de Tamazulapam del Progreso, Oaxaca	17° 41′	97° 35′	1900	8	0	1	0	0	0
4	NE from Santa María Ixcatlán, Oaxaca	17° 52′	97° 10′	2091	20	2	2	0.33684	0.00031	0.4572
5	W from Santa María Ixcatlán, Oaxaca	17° 50′	97°13′	2113	23	0	1	0	0	0
6	W from Santa María Albarradas, Oaxaca	16° 57′	96° 11′	1682	20	0	1	0	0	0
7	“Tecolotiopa”, San Gabriel Casa Blanca, Oaxaca	18° 09′	97° 08′	946	20	0	1	0	0	0
8	“Cerro de las Compuertas”, San Rafael, Coxcatlán, Puebla	18° 11′	97° 08′	989	13	1	2	0.15385	0.00007	−1.1491
**Total**					**128**	**8**	10*	**0.71801**	**0.00078**	**0.3732**

*N*, sample size; *S*, number of polymorphic sites; *h*, number of haplotypes; *Hd*, haplotype diversity; π, nucleotic diversity. All *D’* Tajima test’s values were non-significant. *Total number of haplotypes found in *A. kerchovei*.

### DNA Extraction, Amplification, and Sequencing

Total DNA was extracted by grinding approximately 0.25 g of fresh leaf tissue in liquid nitrogen using a CTAB (2X) extraction protocol (Doyle and Doyle, 1987) and resuspended in 60 μl of ultrapure water (Molecular Biology Reagent; SIGMA).

Three non-coding chloroplast (cpDNA) regions [*psbJ-petA*, *rpl32-trnL* (Shaw et al., 2007) and *trnL-trnF* (Taberlet et al., 1991)] were amplified by polymerase chain reaction (PCR) and sequenced for 136 individuals. The PCR amplifications were carried out in a GeneAmp^®^ PCR system 2700 (Applied Biosystems) in total reaction volumes of 30 μl, containing 25–40 ng of total DNA, 1X of PCR buffer (100 mM Tris-HCL, 500 mM KCl, 10μg/ml gelatin, 1% Triton, 1.5 mg/ml BSA), 1.5 mM MgCl_2_ (for primers *rpl32-trnL* and *trnL-trnF*) and 2 mM MgCl_2_ (for primer *psbJ-petA*), 0.2 mM of each dNTP, 0.3 μM of each primer, and 1 unit of *Taq* DNA polymerase. The cycling conditions consisted of an initial denaturation at 94°C for 5 min, 35 cycles of 94°C for 30 s, 55°C for 30 s (for primers *rpl32-trnL* and *trnL-trnF*) or 55°C for 50 s (for primer *psbJ-petA*), and 72°C for 1 min followed by a final extension at 72°C for 8 min. PCR products were purified and sequenced in the High Throughput Genomic Unit, University of Washington, USA.

The quality of the sequences and the forward and reverse assembly was assessed by direct inspection using the Phrap-Phred, Consed V 19.0 software (Ewing et al., 1998; Gordon et al., 1998). Sequences alignments were made with CLUSTALW (Thompson, 1994) as implemented in BIOEDIT 7.1.3.0 (Hall, 1999). Indels (insertion/deletion) were coded as single base characters to treat them as single events, rather than multiple independent events, and the chloroplast regions were concatenated with DnaSP v5.10.1 (Librado and Rozas, 2009). Sequences were deposited at NCBI GenBank.

### Relationship Among Haplotypes

To assess genetic relationships among haplotypes, we constructed a haplotype network, as implemented in the program TCS v1.21 (Clement et al., 2000) using 95% connection probability limit, treating gaps as single evolutionary events and indels as a fifth state of character. For this analysis, we included sequence data obtained in this study along with sequences from *Agave stricta* and *A. lechuguilla*, from Martínez-Ainsworth (2013) and Scheinvar et al. (2017), respectively.

### Genetic Diversity and Structure

The observed number of haplotypes with (*h*) and without indels (*h*^∗^), haplotype diversity (*Hd*), nucleotide diversity (π), and the Watterson estimator of theta (θ) for each population were obtained using the program DnaSP v5.10.1 (Librado and Rozas, 2009). These summary statistics were re-estimated for the populations of *A. lechuguilla*, reported by Scheinvar et al. (2017).

We used the program Arlequin version 3.5 (Excoffier and Lischer, 2010) to estimate pairwise *F*_ST_ (Weir and Cockerham, 1984) between populations to test for isolation by distance with a Mantel test (Mantel, 1967) and to conduct a molecular analysis of variance (AMOVA) (Excoffier et al., 1992). Finally, with the program PERMUT (Pons and Petit, 1996), we evaluated with 1000 permutations whether there was significant phylogeographic structure by estimating and comparing the differentiation parameters *N*_ST_ and *G*_ST_.

We conducted a spatial analysis of molecular variance (SAMOVA) using Samova version 1.0 to explore the population groupings that maximized the proportion of genetic variance at the total population level (*F*_CT_), without *a priori* assignment of individuals to population groupings (Dupanloup et al., 2002). SAMOVA identifies groups of populations (*K*) that are geographically homogeneous and genetically differentiated from each other while maximizing the proportion of total genetic variance due to differences among groups of locations (*F*_CT_). We explored *K*-values with 100 permutations for each group of populations. The most likely number of groups (*K)* was determined by running the program with different groups of populations which ranged from 2 to 14 groups, choosing those partitions with a maximum *F*_CT_ value, as suggested by Dupanloup et al. (2002). Levels of genetic differentiation among geographic regions and groups identified by SAMOVA were estimated using pairwise *F*_ST_ and 10,000 permutations were used to calculate the corresponding probabilities in Arlequin version 3.5 (Excoffier and Lischer, 2010).

We calculated Tajima’s *D* with DnaSP v5.10.1 (Librado and Rozas, 2009) to infer basic aspects of demographic histories. Tajima’s *D* (Tajima, 1989) statistic is based on the differences between the number of segregating sites and the average number of nucleotide differences. Significant negative *D* (*P* < 0.05) statistic can indicate no neutrality, or population expansion.

### Ecological Niche Modeling

We used Maximum Entropy Modeling (MaxEnt; Phillips et al., 2006) to predict the distribution of *A. kerchovei* for the present day (PRE). MaxEnt is a presence-background algorithm that estimates a species’ potential ecological niche by finding a probability distribution of environmental variables that best describes the occurrence localities, while being able to differentiate between occurrence and background sites (Phillips et al., 2006; Elith et al., 2011; Peterson et al., 2011; Vasconcelos et al., 2012). The predicted distribution was projected into three time periods in the past: the mid Holocene (MH, ∼6 kya); the Last Glacial Maximum (LGM, ∼21 kya), and the Last Inter-Glacial period (LIG, ∼130 kya). These correspond to periods for which global paleo-climate layers are available (Hijmans et al., 2005).

Present day occurrence data for *A. kerchovei* were retrieved from herbarium records and sampled populations, representing 37 unique occurrence localities at a 30 arc-second resolution. The models were built from climate layers obtained from the WorldClim database version 1.4 (Hijmans et al., 2005). We selected five climate layers based on pairwise Pearson correlations. For this, we identified pairs of climate layers with a correlation coefficient greater than 0.8 and then excluded the layer with the highest variance inflation factor (VIF). The five layers correspond to: Annual Mean Temperature (bio 1), Mean Diurnal Range (bio 2), Isothermality (bio 3), Annual Precipitation (bio 12), and Precipitation Seasonality (bio 15). The same five climate layers for the MH and the LGM were obtained from the Community Climate System Model (CCSM; Kiehl and Gent, 2004). The climate layers for the LIG were also obtained from the CCSM (Otto-Bliesner et al., 2006). All climate layers had a 30 arc-second resolution.

We approximated the accessible area (M) for *A. kerchovei* by masking the climate layers with a spatial polygon defined from the terrestrial eco-regions (Olson et al., 2001) inhabited by the species, which was further delimited using a 2° buffer around occurrence localities. The M was extended to include adjacent terrestrial eco-regions, and a 3° buffer for the projection of the models into past climate conditions. Distribution models were predicted using this area to ensure coverage of the temporal range dynamics of the species and to decrease the over-prediction associated to the use of large modeling areas. We used the ‘ENMeval’ package in R (Muscarella et al., 2014; R Core Team, 2019) to identify the best settings for the regularization multiplier (RM) and ‘features’ in MaxEnt; we used the ‘randomkfolds’ method with *k* = 20 over five RM values (0.5, 0.7, 0.9, 1.1, 1.3).

Distribution models were built with 20 replicates using 10,000 random background points, a RM of 0.7, with hinge features only, without extrapolation and no clamping. Models were validated using 25% of the occurrence data using the receiver operating curve (ROC) statistic. We evaluated the replicate models using the area under the receiver operating curve (AUC), where models with values below 0.8 were dismissed. The remaining replicates models were combined to construct the present-day model and then projected into the past climate layers.

We followed the same modeling procedure to generate present-day distribution models for *A. lechuguilla* using 97 unique occurrence localities at a 30 arc-second resolution. These models were projected into the LIG, LGM and MH. In this case, the models were built after selecting eight layers corresponding to Annual Mean Temperature (bio 1), Mean Diurnal Range (bio 2), Mean Temperature of the Driest Quarter (bio 9), Mean Temperature of Warmest Quarter (bio 10), Mean Temperature of Coldest Quarter (bio 11), Annual Precipitation (bio 12), Precipitation of the Driest Month (bio 14), and Precipitation Seasonality (bio 15).

We used the resulting distribution models for *A. kerchovei* and *A. lechuguilla* to visualize the changes in suitable climatic conditions (climate suitability) to which populations may have been subjected since the Last Interglacial period. For this, we extracted the climatic suitability values through time (i.e., LIG, LGM, MH, PRE) for those grid-cells associated with the occurrence localities of sampled populations, being 0 no suitability and 1 the maximum suitability. To account for the possible bias of using a single grid cell to characterize the climate suitability of populations’ localities, we generated 100 replicates of sample localities by adding random noise to the populations’ geographic coordinates within a buffer of ∼10 km^2^ (0.08°) centered on the sampling locality. For each population, we estimated the half sample mode (HSM) of the suitability values across replicates to visualize the changes in climatic conditions through time.

### Genetic Diversity and Environmental Variance

We estimated the variance of environmental variables (i.e., climate and altitude) for sampled populations of *A. kerchovei* and *A. lechuguilla* (Scheinvar et al., 2017). We used the 19 climate layers obtained from the WorldClim database (Hijmans et al., 2005) and the GTOPO30 global digital elevation model (DEM) from the USGS-EROS Data Center. For each of the environmental variables, we extracted the data for those grid-cells associated with the occurrence localities of sampled populations. As mentioned above, we accounted for the possible bias of using a single grid cell to characterize the environment of populations by generating 100 replicates of sampled localities.

Populations of *A. lechuguilla* were assigned into four different groups according to their geographic location and genetic composition (Scheinvar et al., 2017), whereas populations of *A. kerchovei* were treated as a single group (Table 1). We approximated the environmental variance by performing a Principal Component Analyses on populations’ environmental conditions and estimating the variance for the first two principal components. We tested the correlation between environmental variance and genetic diversity (i.e., nucleotide and haplotype diversity) among the five groups of populations using simple linear regression. For this, we used the components of the environmental PCA as predictors and the indices of genetic diversity as response variables.

## Results

### Genetic Diversity and Structure

The combination of the three non-coding chloroplast (cpDNA) regions [*psbJ-petA*, *rpl32-trnL* (Shaw et al., 2007)] and *trnL-trnF* (Taberlet et al., 1991) resulted in sequences 2,188 bp long. In total, we found eight segregating sites and eight indels, resulting in ten haplotypes *for A. kerchovei* (nine when not considering indels). Total haplotype diversity (*Hd*) was 0.718, but the average per population diversity was far lower (*Hd* = 0.107), indicating strong genetic differentiation among populations (Table 1). Total nucleotide diversity (π) was low (0.00078), with most populations having null nucleotide diversity.

The haplotype network was well resolved with ten haplotypes for *A. kerchovei* (Figure 1B). The only haplotype shared between more than two populations was haplotype 2, shared among four and was also the haplotype with the highest frequency. The remaining nine haplotypes were found to be exclusive to single populations, with five populations being fixed for a particular haplotype (Figure 1A). The network has a star-like shape, with the most common haplotype (h2) at the center of the network; from this haplotype, two singe haplotypes are derived (3 and 4), that belong to population 1 (P1) and three haplogroups, one consisting of haplotypes 1 and 6, another one with haplotypes 10, 8 and 7, where there is one missing mutation, and a third one that comprises haplotypes 2 and 6 with seven missing mutations, which belong to populations located outside the Tehuacán Valley (P2 and P6), being population 6 the most isolated. From the central and most common haplotype (2), the haplotypes found in *A. stricta* and *A. lechuguilla* are also derived (Figure 1B).

The prevalence of private haplotypes within populations resulted in a high genetic differentiation (*F*_ST_ = 0.928). According to the PERMUT analysis, there are no significant differences comparing *N*_ST_ (0.943) vs. *G*_ST_ (0.880), suggesting lack of phylogeographic structure. Therefore, a Mantel test was not significant (data not shown), which suggests that there is no evidence of isolation by distance in *A. kerchovei*. Tajima’s *D*, although positive, 0.37320, was not significant (*p* = 0.704).

In the AMOVA, the highest percentage of variation (92.80%) was explained by differences among populations, whereas only 7.20% was found within populations (Table 2). Total genetic differentiation (0.928) indicates a high genetic structure. Therefore, most of the variation within the species can be explained by differences between populations, rather than the difference within populations. In accordance, a SAMOVA analysis suggested six groups with a value of *F_CT_* = 0.915. One cluster is composed of three populations (1, 7 and 8), in the North, while the rest of the groups are conformed only by one population each (Figure 1A).

**TABLE 2:** Results of analysis of molecular variance (AMOVA) of the analyzed populations of *A. kerchovei* for cpDNA.

Source of variation	d.f.	Sum of squares	Variance components	Percentage of variation
Among populations	7	243.430	2.214*	92.80
Within populations	120	20.623	0.172	7.20
Total	127	264.062	2.385	
FST* = 0.928				

*P < 0.01, d.f., degrees of freedom.

### Ecological Niche Analysis

**FIGURE 3:**
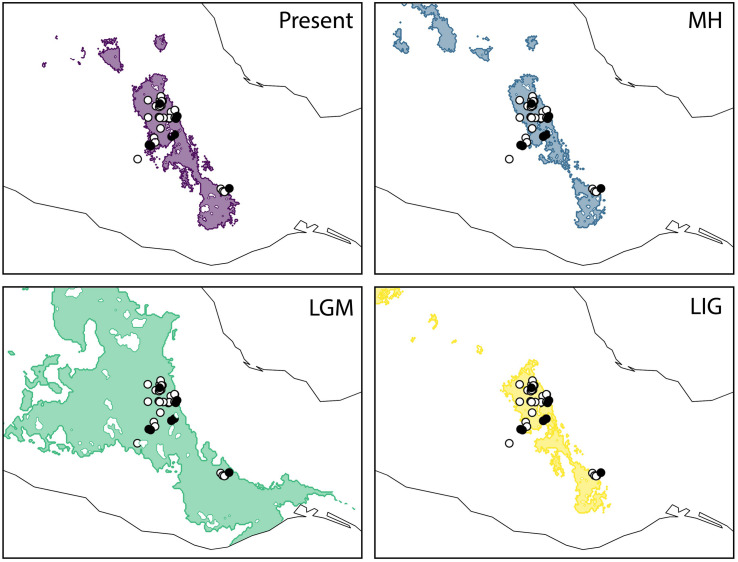
Geographical projections of the ecological niche model for *Agave kerchovei* for the present-day, the middle Holocene (MH, ∼6 kya), the Last Glacial Maximum (LGM, ∼21 kya), and the Last Interglacial (LIG ∼110 kya). Shaded areas in past models represent the projected areas potentially inhabited by the species. Black circles represent the populations sampled for the present study (eight populations). White circles represent the occurrence records used to construct the ecological niche models (37 records).

The predictive performance of the bioclimatic models was adequate with an AUC > 0.82 across replicates. The projected distribution for *A. kerchovei* during the LIG shows the most restricted distribution across all the time periods analyzed, where ideal climate conditions for the species were geographically restricted to an area equivalent to 75% of the present-day distribution (Figure 3). Subsequently, according to our models, the LGM witnessed a significant geographical expansion in the ideal climate conditions for *A. kerchovei*, which were broadened by 522% relative to the present-day distribution. The projected models then predicted a geographical contraction for *A*. *kerchovei* during the MH (93% relative to the present-day), with this geographical extent remaining stable ever since. Our models revealed two main areas that have remained more or less stable and isolated from each other: (1) the Tehuacán Valley in the North, and (2) the Central Valleys of Oaxaca in the South.

**FIGURE 4:**
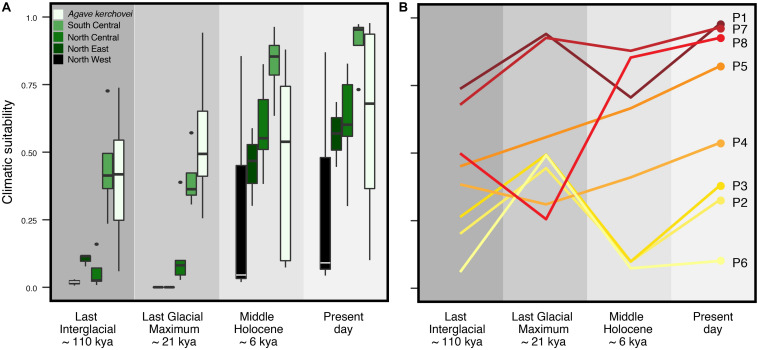
Climatic suitability through time estimated across sampled populations of *Agave kerchovei* and *A. lechuguilla* using the projections of the species’ ecological niche models. **(A)** Distribution of climatic suitability values through time within the five groups of populations of *Agave kerchovei* and *A. lechuguilla*. **(B)** Climatic suitability values through time for the eight sampled populations of *A. kerchovei*. Population names as in Table 1.

Accordingly, we characterized the changes in suitable climatic conditions (climate suitability) to which the geographic regions corresponding to present-day sampled populations may have been subjected since the LIG. We found that the climatic suitability varies significantly through time (i.e., LIG, LGM, MH, PRE) among the occurrence localities of each sampled population (Figure 4B). Interestingly, most occurrence localities show an improvement in the suitability values during the LIG–LGM transition, with this improvement trend continuing into the MH and present-day, but only for those populations within the core areas of the Tehuacán Valley (populations P1, P4, P5, P7 and P8; Figure 4B). Also, our models show that the three most isolated populations – which lie outside the Tehuacán Valley (P2, P3 and P6, Figure 1A) and harbor the most divergent haplotypes – experienced a dramatic decline in climatic suitability toward the MH (Figure 4B).

To explore whether the detected patterns are similar in other *Agave* species with wider distribution we characterized the temporal variation in climatic suitability in the widespread *A. lechuguilla* from the Chihuahuan Desert, finding that the temporal changes in climate suitability are different from those observed for *A. kerchovei* (Figure 4A). In *A. lechuguilla*, we observed a drastic increase in the climate suitability in occurrence localities during the LGM–MH transition and into the present-day for northern populations of *A. lechuguilla*, whereas the southern populations showed a climate suitability trend resembling *A. kerchovei* (Figure 4A). In this context, we suggest that the Tehuacán Valley, along with the southernmost portion of the Chihuahuan Desert, have been regions with relatively stable climatic conditions suitable for the survival of *Agave* species through the last 110,000 years, explaining, at least in part, the high *Agave* species diversity in these areas (Scheinvar et al., 2017).

### Genetic Diversity and Environmental Variance

The first two components of the PCA on environmental variables for *A. kerchovei* explained 46.18% and 21.07% of the total climatic variance, respectively (data not shown). The first PCA was most strongly positively correlated with annual precipitation, summer precipitation and altitude, whereas it showed the most negative correlation with summer temperature. On the other hand, the second PCA was most positively correlated with winter precipitation, and negatively with winter temperature and precipitation seasonality (data not shown).

**FIGURE 5:**
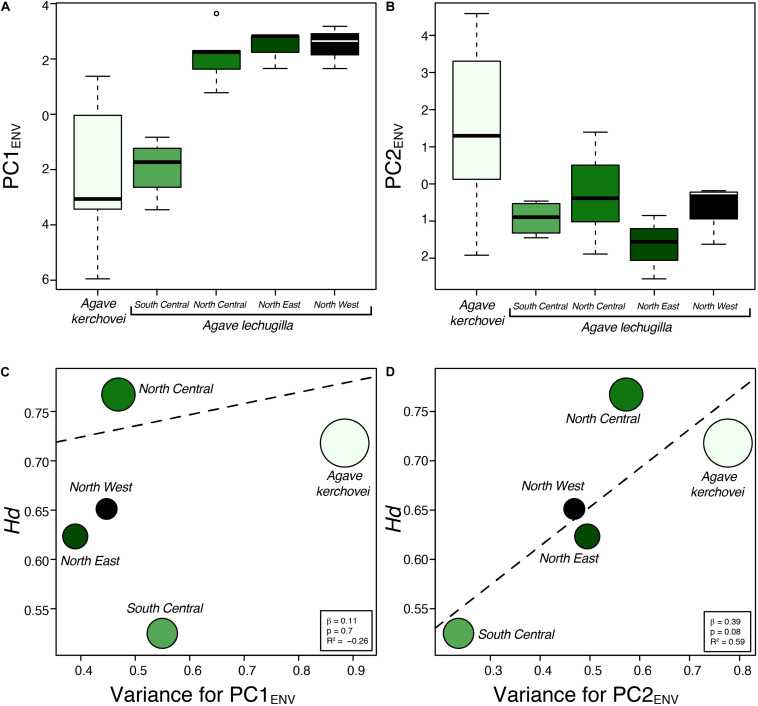
Environmental variance observed across sampled populations of *Agave kerchovei* and *A. lechuguilla*. **(A,B)** Environmental values in the two species of *Agave* obtained from the scores for the first two principal components of the environmental principal component analysis (*PC1*_ENV_, *PC2*_ENV_). **(C,D)** Relationship between environmental variance (*PC1*_ENV_ and *PC2*_ENV_) and haplotype diversity estimated for the five groups of populations. Dashed line depicts linear regression models. Circle size is proportional to sample size (*n*).

**FIGURE 6:**
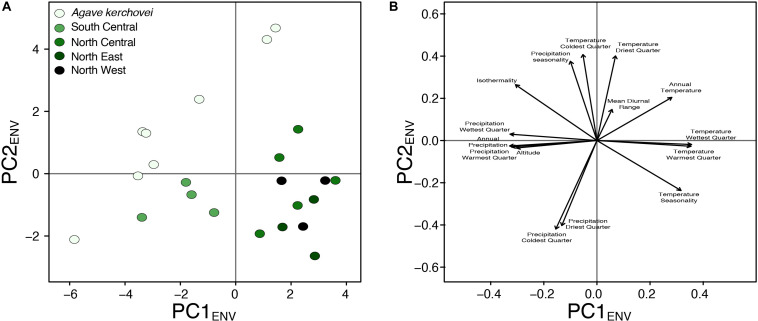
Principal component analyses of environmental preferences for *Agave kerchovei* and *A. lechuguilla*. **(A)** Scores plot, and **(B)** loading plot for the first two principal components (*PC1*_ENV_, *PC2*_ENV_).

We also compared the environmental variance within groups of sampled populations of *A. kerchovei* and *A. lechuguilla* (Scheinvar et al., 2017; Scheinvar, 2018) with a principal component analyses on populations’ environmental (PCA_ENV_) conditions, and estimating the variance for the first two principal components (Figures 5A,B), which jointly accounts for 69.6% of the variance (47.4% and 22.2%, respectively; Figure 6). Populations of *A. kerchovei* and *A. lechuguilla* are broadly ordered in a South–North direction along the first two principal components (Figure 6A). The PC1_ENV_ was most strongly associated with temperature and precipitation during the summer months (i.e., the Wettest and Warmest Quarters), whereas the PC2_ENV_ was most strongly associated with temperature and precipitation during the winter months (i.e., the Driest and Coldest Quarters) (Figure 6B). For these two principal components (PC1_ENV_ and PC2_ENV_), we obtained each population’s scores and estimated the variance within geographically defined groups of populations (Figure 6A). The range of values within groups of populations for PC1_ENV_ and PC2_ENV_ shows a pattern where more environmental variance exists among populations of *A. kerchovei* than within geographically defined groups of populations of *A. lechuguilla* (Figures 5A,B).

The linear regression models showed no association (*b* = 0.11, *p* = 0.7) between levels of haplotype diversity within groups of populations and their variance along PC1_ENV_ (Figure 5C). Nonetheless, we found a positive association (*b* = 0.39, *p* = 0.08, *R*^2^ = 0.59) between the levels haplotype diversity within groups of populations and their variance along PC2ENV (Figure 5D). Sample size was not correlated with haplotype diversity (*b* = 0.001, *p* = 0.47) nor with estimates of environmental variance along PC2_ENV_ (*b* = 0.005, *p* = 0.17), but it was correlated with environmental variance along PC1_ENV_ (*b* = 0.006, *p* = 0.02). The high environmental variance along PC2_ENV_ observed among populations of *A. kerchovei* – which is associated with variance in winter temperatures and precipitation (Figure 6B) – is reflected in higher levels of total genetic diversity than those observed for most of the groups of populations of *A. lechuguilla*. This is also probably facilitated by the geographical isolation of populations within the Tehuacán Valley (Figure 1A). However, nucleotide diversity did not show a significant correlation with environmental variance.

## Discussion

*Agave kerchovei* is a species with a restricted distribution, with populations mainly in the Tehuacán Valley. As occurs in many rare or endemic species with few and usually small populations, some of its populations are completely depleted of genetic variation and genetic structure is high, which may be a consequence of demographic phenomena such as inbreeding, genetic bottlenecks or drift (Hamrick and Godt, 1990; Nybom and Bartish, 2000; Gibson et al., 2008). Pleistocene glacial cycles had an important role in the distribution of *A. kerchovei*, and climatic variability appears to have had a significant effect on the levels of genetic diversity and differentiation among populations. Overall, in terms of climate, the species’ core distribution area within the Tehuacán Valley appears to have remained relatively stable over the last ∼6 kya, whereas peripheric populations outside the Valley, which apparently are genetically isolated, have been subjected to more climatic instability.

### Genetic Diversity and Structure

There have been several studies in the population genetics of different *Agave* species. Earlier studies were reviewed in Eguiarte et al. (2013). In general, agaves are rich in genetic variation, and usually genetic differentiation is low among populations, but there is a wide variation among species (e.g., Byers et al., 2014; Parker et al., 2014; Félix-Valdez et al., 2016; Trejo et al., 2016; Figueredo-Urbina et al., 2017). For *A. kerchovei*, considering its restricted distribution, the species shows higher levels of chloroplast genetic variation (*Hd* = 0.718) than those estimated for *A. stricta* (*Hd* = 0.524), which is an endemic of the Tehuacán Valley (Martínez-Ainsworth, 2013). This might be related to significant biological and ecological differences between the species, with *A. kerchovei* inhabiting regions of more topographic complexity (Gentry, 1982), and hence climate variability, than those inhabited by *A. stricta*, but a detailed study on the differences of the distribution and ecology between this two species would be necessary to deepen in the causes of this observation. Nevertheless, low levels of genetic diversity were found within populations of *A. kerchovei*, with five of the eight populations analyzed having no genetic variation. Although these two *Agave* species show different levels of genetic diversity, it is worth noting that the two show lower levels of chloroplast diversity than one wide ranging *Agave* species from the Chihuahuan Desert, *A. lechuguilla* (*Hd* = 0.931) (Scheinvar et al., 2017; Scheinvar, 2018), while *A. kerchovei* has similar values to another wide ranging *Agave* species from the Chihuahuan Desert, *A. striata (Hd* = 0.713), the sister species of *A. stricta* (Martínez-Ainsworth, 2013).

Genetic differentiation was high in *A. kerchovei* (*F_ST_* = 0.928, *G_ST_* = 0.824). High genetic structure using similar chloroplast sequences was also found in *A. striata* (*F*_ST_ = 0.929, *G_ST_* = 0.697; Martínez-Ainsworth, 2013) and *A. stricta* (*F*_ST_ = 0.944, *G*_ST_ = 0.898; Martínez-Ainsworth, 2013), but it was lower in *A. lechuguilla* (*G_ST_* = 0.780; Scheinvar, 2018; Scheinvar et al., 2017).

Throughout most of the distribution of *A. kerchovei* differentiation among populations appears to be very high, with the exception of the northernmost populations (P1, P7, P8) (Figure 1A), usually harboring a single, unique (private), haplotype. This could be the result of a combination of ecological and historical factors, including changes in climate and the rough and complex topography of the region, generating different conditions that restrict the types of vegetation within the altitudinal ranges (Valiente-Banuet et al., 2000, 2009). In this case, genetic differentiation among populations would result due to restricted gene flow, small effective population sizes and strong adaptation to local abiotic and biotic conditions. Thus, populations in most of the cases seem to be evolving locally, which could suggest a process of local adaptation. For instance, Brena-Bustamante (2012) found that percentage of germination in the field is different for each of the populations studied (P7, San Gabriel, 41.4% under shadow and 6.6 under light conditions versus 19% and 5.4% respectively in P8, San Rafael), suggesting population differentiation. In addition, vegetation cover and species composition are very different for each population, despite being very near (∼5 km, Brena-Bustamante et al., 2013) and belonging to the same broad type of vegetation (tropical deciduous forest), suggesting that the environment is different, and in consequence local adaptation could also differ.

Strong genetic differentiation among populations of *A. kerchovei* is further supported by the absence of a clear phylogeographic signal, the absence of isolation by distance among populations and the fact that some populations consist of very few individuals. Another important biological factor that may be relevant to explain the genetic patterns observed in *A. kerchovei* is clonal reproduction, possibly leading to patches of genetically identical individuals. Accordingly, Brena-Bustamante (2012) found evidence of sexual and asexual reproduction in *A. kerchovei*. Clonal propagation would reduce the number of genetic individuals in a population (Honnay and Jacquemyn, 2008; Vallejo-Marín et al., 2010) and would explain at least in part why most sampled populations only harbor one single chloroplast haplotype. In order to better asses the importance of asexual reproduction, it will be relevant to use in the future nuclear DNA to better disentangle the roles of seed and clonal reproduction.

### Evolutionary History

It has been suggested that the Pleistocene was an important period for the diversification of *Agave* (Scheinvar et al., 2017; Scheinvar, 2018). Accordingly, the divergence of *A. kerchovei* has been dated to the Pliocene-Pleistocene transition (∼2.6 Mya) (Scheinvar et al., 2017). The main inferred climate changes during this period in North America, were in mean temperatures, which are believed to be one of the main factors promoting range fragmentations/expansions and population isolation in plant species (Ramírez-Barahona and Eguiarte, 2013; Scheinvar et al., 2017). Accordingly, we inferred a historical range dynamic in *A. kerchovei* over the last 110,000 years, involving range expansion and contraction. More specifically, our results suggest that during the LIG, which in Central Mexico has been characterized as a more humid and warmer period (Metcalfe, 2006), *A. kerchovei* appears to have suffered its maximum range contraction (Figure 3), possibly surviving within isolated areas throughout the Tehuacán Valley. Over this period, arid adapted species decreased their distribution area and became isolated into smaller populations or refugia, increasing their population differentiation (Scheinvar et al., 2017). It has been shown that physiological and morphological strategies allow Agaves to survive under extreme temperature conditions (Nobel and Smith, 1983; Martinez-Rodriguez et al., 2019). Afterward, the species appears to have experienced a significant range expansion during the LGM (∼21 kya), when the climate became drier and colder (Caballero et al., 2010), with a contraction in the MH and remaining stable until the present.

However, changes in climatic suitability appear to have been heterogeneous across the distribution of the species, showing that localities from the area of the Tehuacán Valley have exhibited a continuous improvement in the suitability values in the last 110,000 years, while in contrast, the three most isolated populations (P2, P3, and P6, Figure 1A), which also happen to harbor the most divergent haplotypes, experienced a dramatic decline in climatic suitability toward the MH (Figure 4B). In other words, populations in the core area appear to have experienced less changes than populations in the periphery, resulting in two main areas that have remained more or less isolated for *A. kerchovei*: (1) the Tehuacán Valley in the North and (2) the Central Valleys of Oaxaca in the South. This is in accordance with our genetic analysis of the distribution of variation in *A. kerchovei*, as the northern populations share a common haplotype, while the rest of the populations have unique haplotypes, resulting in high genetic structure, as shown by the analysis of AMOVA and SAMOVA. In the latter, almost every location is designated as a separate group. This could be attributed to genetic drift and local differentiation and adaptation, as for instance different microenvironmental conditions are found within each area in this region of Mexico (Valiente-Banuet et al., 2000, 2009), further promoting differentiation.

This differentiation could represent a process of incipient speciation, particularly regarding population P6, which is the most isolated at the eastern and southernmost extreme of its distribution. In the northern area, conformed of populations P1, P7, and P8, haplotype 2 is the most common and according to the network it can be considered as the ancestral haplotype, suggesting this area could be the ancestral area of the distribution.

When we compare temporal variation in climatic suitability in *A. lechuguilla* from the Chihuahuan Desert and in *A. kerchovei*, we can appreciate temporal changes appear to be very different in northern populations of *A. lechuguilla*, but not in the southernmost populations of the species, which show a climate suitability trend resembling the one observed for *A. kerchovei.* We suggest that the Tehuacán Valley, together with the southernmost portion of the Chihuahuan Desert, have been regions with climatic conditions suitable for the survival of *Agave* species through the last 110,000 years.

## Conclusion

Our results suggest that Pleistocene climate fluctuations and the resulting contraction and expansion of *A. kerchovei* populations have led to changes in population sizes in which both genetic drift and the subsequent expansion of the distribution were probably important factors in generating the currently observed genetic structure. The high environmental variance observed among populations of *A. kerchovei* is reflected in high levels of among population differentiation, probably due to the geographical isolation of populations within and outside the Tehuacán Valley. Thus, topographic complexity through its effects on climatic variability appears to have a significant impact on the levels of genetic diversity and structure among populations.

We suggest that in *A. kerchovei* we have a core group of populations in the Tehuacán Valley, and peripheric populations that seem to be evolving independently, as shown also by the haplotype network. In this sense, we think that *A. kerchovei* is basically an endemic species from the Tehuacán Valley, and that populations outside the Valley are in the process of incipient speciation.

## Data Availability Statement

Sequences were deposited at NCBI GenBank (KX444126–KX444129, KX444111–KX444115, MT513760–MT513762, and MT511772–MT511781).

## Author Contributions

EA-P contributed to laboratory work, genetic analysis, and drafting the manuscript. JP-L contributed to laboratory work, genetic analysis and the design of some figures. SR-B contributed to fieldwork, data analysis, and helped in drafting and correcting sections of the manuscript, and designing some figures. ES contributed with genetic analysis. RL-S contributed with logistics and ideas for the design of the project. LE project leader, designed and coordinated the project, logistics, drafted and corrected the manuscript. All authors contributed to the article and approved the submitted version.

## Conflict of Interest

The authors declare that the research was conducted in the absence of any commercial or financial relationships that could be construed as a potential conflict of interest.

## Footnotes

1 http://museum2.utep.edu/chih/chihdes.htm

2 http://legacy.tropicos.org/

3 http://www.theplantlist.org

4 https://www.gbif.org
